# Identification of a non-purple tartrate-resistant acid phosphatase: an evolutionary link to Ser/Thr protein phosphatases?

**DOI:** 10.1186/1756-0500-1-78

**Published:** 2008-09-04

**Authors:** Kieran S Hadler, Thomas Huber, A Ian Cassady, Jane Weber, Jodie Robinson, Allan Burrows, Gregory Kelly, Luke W Guddat, David A Hume, Gerhard Schenk, Jack U Flanagan

**Affiliations:** 1 School of Molecular and Microbial Sciences, The University of Queensland, St. Lucia, 4072, Australia; 2 Cooperative Research Centre for Chronic Inflammatory Disease, Institute for Molecular Bioscience, The University of Queensland, St. Lucia, 4072, Australia; 3 ARC Special Research Centre for Functional and Applied Genomics, Institute for Molecular Bioscience, The University of Queensland, St. Lucia, 4072, Australia

## Abstract

**Background:**

Tartrate-resistant acid phosphatases (TRAcPs), also known as purple acid phosphatases (PAPs), are a family of binuclear metallohydrolases that have been identified in plants, animals and fungi. The human enzyme is a major histochemical marker for the diagnosis of bone-related diseases. TRAcPs can occur as a small form possessing only the ~35 kDa catalytic domain, or a larger ~55 kDa form possessing both a catalytic domain and an additional N-terminal domain of unknown function. Due to its role in bone resorption the 35 kDa TRAcP has become a promising target for the development of anti-osteoporotic chemotherapeutics.

**Findings:**

A new human gene product encoding a metallohydrolase distantly related to the ~55 kDa plant TRAcP was identified and characterised. The gene product is found in a number of animal species, and is present in all tissues sampled by the RIKEN mouse transcriptome project. Construction of a homology model illustrated that six of the seven metal-coordinating ligands in the active site are identical to that observed in the TRAcP family. However, the tyrosine ligand associated with the charge transfer transition and purple color of TRAcPs is replaced by a histidine.

**Conlusion:**

The gene product identified here may represent an evolutionary link between TRAcPs and Ser/Thr protein phosphatases. Its biological function is currently unknown but is unlikely to be associated with bone metabolism.

## Background

Purple acid phosphatases (PAPs) are a diverse group of metalloenzymes that catalyse the hydrolysis of phosphate esters and anhydrides [1]. PAPs are resistant to inhibition by L(+)-tartrate, a potent inhibitor of other acid phosphatases, and as such are also known as tartrate-resistant acid phosphatases (TRAcPs; alternative names include ACP5, TRAP) [1]. They contain a bimetallic active site comprising seven coordinating amino acids that are conserved in all PAP isoforms identified to date [1,2]. One metal site is invariably an Fe(III) and the characteristic purple color of TRAcPs arises from a tyrosine to Fe(III) charge transfer transition [1]. The other site contains a divalent metal ion where M(II) = Fe, Zn or Mn depending on the source of the protein [1-5]. The X-ray crystal structures of TRAcPs from several sources, including human, pig, red kidney bean and sweet potato have been determined [6-9]. Notably, although their sequence identity is only < 20%, these enzymes have a common core structure with five motifs that contain the invariant seven metal coordinating amino acids in the catalytic site [2].

TRAcPs have been isolated from a range of plants, mammals and fungi, and TRAcP-like sequences have also been identified in a number of bacteria [1]. Structural and biochemical characterisation of the TRAcPs from the red kidney bean, *Phaseolus vulgaris*, and sweet potato, *Ipomoea batatas*, have demonstrated their existence as homodimers with subunits of ~55 kDa [1,5]. The plant isoforms may also exist as heterodimers of 57 and 63 kDa subunits [1]. The catalytic centres of the red kidney bean, soybean and one isoform from sweet potato enzyme contain an Fe(III)-Zn(II) complex, whereas Fe(III)-Mn(II) is present in the other sweet potato form [1]. Plant TRAcPs have been shown to exhibit an amino acid sequence similarity of > 70% [2]. Mammalian TRAcPs have been characterised from multiple species including human, pig, cow, mouse and rat, and all exist as monomers of ~35 kDa, that share > 80% sequence identity and contain redox-active Fe(III)-Fe(III)/Fe(II) centers [2,10]. A number of distinct TRAcP isoforms were identified in plants and bacteria, clearly illustrating the existence of multiple TRAcP genes in different kingdoms [1,2]. This is further supported by the existence of a plantlike TRAcP in animals [1].

The biological roles for TRAcPs are diverse and species-dependent. Evidence has accumulated that links the mammalian enzymes to bone metabolism and bacterial killing, while plant enzymes maybe have a function in phosphate metabolism [10]. Specifically, it could be shown that in transgenic mice the level of TRAcP expression correlates with the extent of bone resorption; TRAcP-knockout mice display symptoms characteristic for osteoporosis, while mice overexpressing TRAcP display an osteoporotic phenotype [11,12]. TRAcP is a major histochemical marker for the diagnosis of bone-related diseases, and elevated serum concentrations of are also observed in patients with Paget's disease, osteosarcoma, breast and prostate cancer. Due to its role in bone resoption TRAcP has become a target for the development of anti-osteoporotic chemotherapeutics [13].

The design of such chemotherapeutics necessitates a high degree of specificity, in particular since enzymes closely related to TRAcPs may function in completely different roles in metabolism. We have thus extended our previous work on investigation of TRAcP and TRAcPlike protein content in animal genomes and identified a new gene product that is a remote homolog to both TRAcPs and Ser/Thr protein phosphatases.

## Findings

### Homolog identification and characterisation

The human TRAcP (ACP5) sequence (accession number NP_001602; unless stated otherwise accession codes are NCBI reference sequence numbers) was used to perform a five iteration PSI-BLAST search of the non-redundant database (the search conditions were the same as described previously [14]). This search identified a distantly related human sequence with the accession number NP_060810, that had 15% sequence identity and 29% similarity to the original acp5 query sequence. Related gene products from other eukaryotes were identified in the NCBI Homologene database  and ENSEMBL resources , and included *Bos taurus *(NP_001026941) *Pan troglodytes *(XP_001145620), *Canis familiaris *(XP_536969), *Mus musculus *(NP_666179), *Rattus norvegicus *(NP_001013985), *Gallus gallus *(XP_414732) and *Plasmodium falciparum *(XP_001348209) indicating that this new gene product is evolutionarily conserved. The new human sequence was used to query the nr database to search for the closest relative with known structure, and identified the catalytic domain of TRAcPs from red kidney bean (*P. vulgaris*), 4KBP [6], and sweet potato (*I. batatas*), 1XZW [8]. Although sequence identities were low (18% across 246 residues as determined by PSI BLAST analysis) the E-values for the profile based search were 2 × 10^-72 ^and 8 × 10^-68 ^respectively, clearly indicating a significant relationship between these proteins and the novel sequence. Alternative transcripts for the mouse and human sequences were also included.

**Figure 1:**
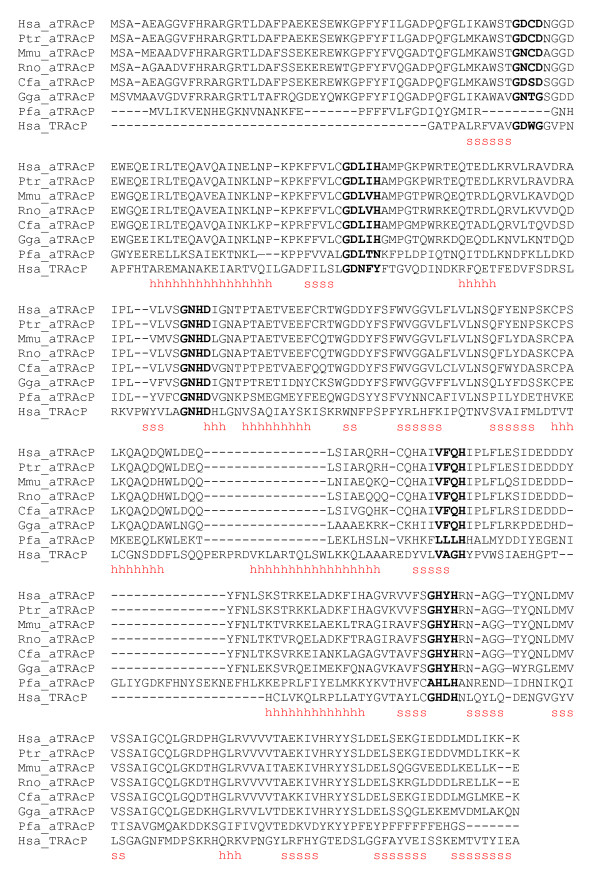
Multiple sequence alignment of the eukaryotic aTRACP gene products. Alternative transcripts identified in mouse and human are appended. The five motifs containing the seven metal coordinating residues observed in TRAcPs have been superimposed on the alignment (Me_motif_1XZW). The position is based on the WURST generated pairwise alignment between the human gene product and the sweet potato enzyme. For comparison the sequence of human TRAcP (*Hsa*_TRAcP) is also included, together with its secondary structure elements (h: helix; s: β-sheet; sheet 14 is omitted). Species identifiers: *Hsa, Homo sapiens; Ptr, Pan troglodytes; Mmu, Mus musculus; Rno, Rattus norvegicus; Cfa, Canis familiaris; Bta, Bos Taurus; Gga, Gallus gallus; Pfa, Plasmodium falciparum*.

The sequences identified here were aligned using T-coffee, as shown in Figure 1[15]. As can be seen from the alignment, the sequence conservation across species is high, with the *G. gallus *gene product 70% identical to the human, dropping to approximately 32% for the *P. falciparum*. Illustrated within this figure are five motifs, that are reminiscent of known TRAcP sequences that contain the metal coordinating ligands and can be represented by the patterns (1) DxG, (2) GDx2Y, (3) GNH [E, D], (4) Vx2H, (5) GHxH, where x represents any amino acid [2]. The notable difference is the concerted substitution of the Tyr in motif 2 by a His in the new sequences. This Tyr is essential for the purple color and the presence of an iron in the trivalent oxidation state in the active site of TRAcPs (see above), whereas a His residue in this position is seen in other binuclear metallohydrolases including the Ser/Thr protein phosphatases [1]. Based on the identity of the amino acid residues that are likely to line the active site pocket in the novel gene product it is probably that this protein is a non-purple enzyme with phosphatase activity. We have thus labelled it *Hsa*_aTRAcP (aTRAcP: alternative TRAcP).

**Figure 2:**
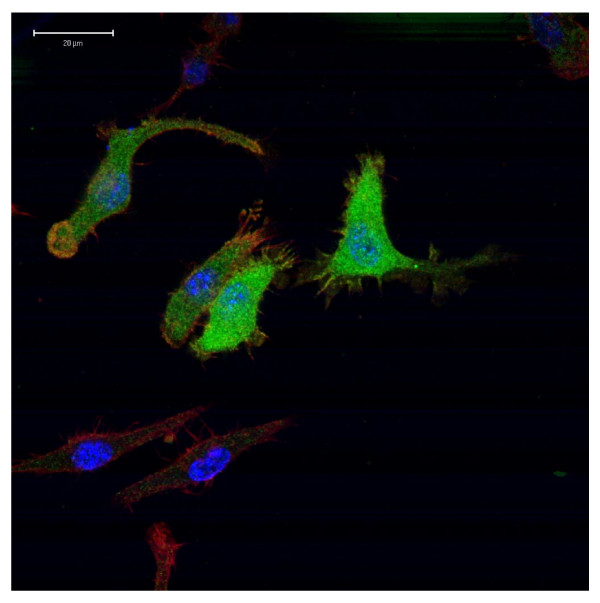
Subcellular localisation of *Hsa*_aTRACP. Immunofluoresence staining of RAW264.7 cells transfected with a *Hsa*_aTRACP-V5 using the Alexa488 goat anti-mouse antibody (Invitrogen) in combination with the mouse anti-V5 IgG2a (Serotec) and visualised using Alexa594 Phalloidin stain (Invitrogen). DNA was stained with DAPI (Roche). Evident is the diffuse cytoplasmic distribution of *Hsa*_aTRACP.

A large number of phosphatases are present in eukaryotic organisms. Many acid phosphatases, including the mammalian TRAcPs, are lysosomal enzymes and have signal peptides and lysosomal targeting sequences. No such sequences are evident in this new protein. To test the location within the cell, we constructed a mammalian expression plasmid with a V5 epitope tag (Figure 2). When this tagged protein is expressed in RAW264 macrophages, the predominant location is diffuse cytoplasmic with no evident membrane association. It is thus likely that the biological role of *Hsa*_aTRAcP is different from that of its purple counterparts.

The intron-exon structure of the gene encoding this putative phosphatase (C530044N13Rik; ENSEMBL Gene ENSMUSG00000065979) comprises only 4 exons, spread over more than 100 kb of genomic DNA, a structure that is widely conserved in vertebrates. From analysis of RIKEN transcriptome data for the mouse homolog (GeneID 223978) using the CAGE analysis viewer , it is evident that the gene locus is actively transcribed in almost all tissues examined, including embryonic tissue as well as adult liver, lung, macrophages and neural tissue with little variation in CAGE Tag frequency (an index of gene expression). The promoter is conserved between mice and human, is relatively GC-rich, and initiates transcription at multiple sites in a 100 bp window around the site of the largest CAGE tag cluster, features consistent with a possible "housekeeping" gene function.

### Structure prediction of *Hsa*_aTRAcP

To further assess the novel sequences as non-purple binuclear metallohydrolases, a structural model of *Hsa*_aTRAcP was constructed by comparative modelling using the sweet potato TRAcP coordinates [8]. The only proteins with known structure identified from the PSI-BLAST search were the plant TRAcPs and the phosphodiesterase from *Mycobacterium tuberculosis *(Rv0805, 2HY1 [16]). An additional phosphodiesterase was identified from *Enterobacter aerogenes *(2dxn [17]), using the threading based approach mGenThreader [18]. The prediction reliability scores for the bacterial diesterases were 114.5 and 105.8, respectively, with corresponding p-values (probabilities of false positives) of 1 × 10^-10 ^and 1 × 10^-9^. These values are similar to those obtained for the closest TRAcP homologue, the enzyme from sweet potato (1XZW, with a reliability score of 87.3 and a p-value of 8 × 10^-8^. This strongly implies that *Hsa*_aTRAcP will adopt a fold similar to these proteins.

**Figure 3:**
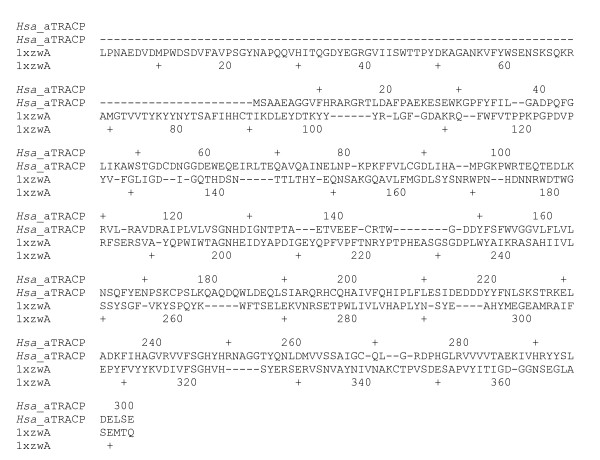
Sequence to structure alignment generated by WURST for the query sequence, *Hsa*_aTRACP (Accession number Hsa_NP_060810), and the template structure from sweet potato TRAcP, pdb code 1XZW.

Due to the low sequence identity between the *Hsa*_aTRACP and the sweet potato enzyme, the sequence-to-structure alignment method WURST was used to generate an alignment (Figure 3), that was subsequently used as input into MODELLER for coordinate generation.

**Figure 4:**
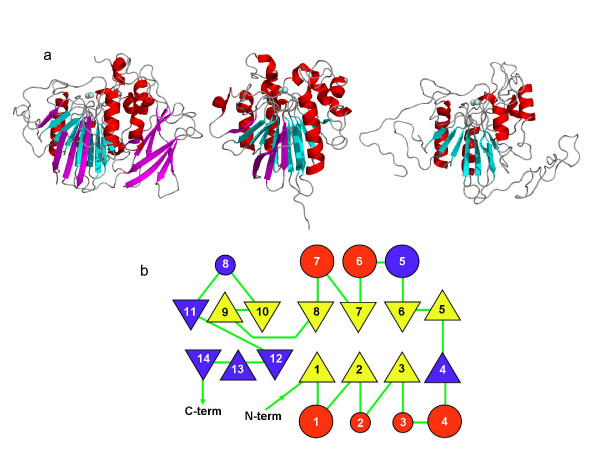
(A) Cartoon diagrams of the high molecular weight sweet potato TRAcP template structure, 1XZW, the low molecular weight human TRAcP (Acp5), 1WAR, and the query sequence, *Hsa*_aTRACP. Secondary structure elements not shared between the known structures and *Hsa*_aTRACP model are colored magenta. The Fe(III) Fe(II) atoms in the active site are represented as spheres. (B) Comparison of topologies for the low molecular weight human TRAcP and *Hsa*_aTRACP. Secondary structure elements that are common to both proteins are colored yellow (for β-strands) and red for (α-helices). Secondary structure regions that are only observed in human TRAcP are colored blue. For *Hsa*_aTRAcP the regions of secondary structureare S1(residues 49–51), H1 (67–74), S2(85–87), H2(102–110), S3(121–122), H3(127–130), H4(141–144), S5(151–155), S6(158–162), H6(180–191), S7(199–203), H7(225–238), S8(242–245), S9(256–258) and S10(280–284). For human TRAcP (*Hsa*_TRAcP; see also Fig. 1) the regions of secondary structure are S1(5–10), H1(24–39), S2(44–47), H2(64–68), S3(83–85), H3(91–93), H4(96–104), S4(109–110), S5(116–121), S6(128–133), H5(136–145), H6(157–173), S7(178–182), H7(197–209), S8(214–217), S9(223–227), S10(233–237), H8(250–252), S11(258–262), S12 (270–276), S13 (280–287) and S14(292–299).

In general, TRAcPs typically consist of two β-sheets each with seven strands and flanked by two α-helices, and is illustrated in Figure 4A for both the plant template structure, and the smaller mammalian isoform. Five of the beta strands position loop structures that contain metal coordinating ligands [7]. Inspection of the model indicates that *Hsa*_aTRACP has both sheets conserved, however, one has only six strands and the second, three (Figure 4A and 4B). Notably, the strands contributing the metal coordinating ligands are all conserved in *Hsa*_aTRACP. Furthermore, loops contributing to substrate binding in TRAcPs and TRAcP-like proteins are partially conserved in our model structure. The I-TASSER server was used to produce alternative predicted structures, and gave a top ranked model that appeared to be a composite of the plant PAP and bacterial Rv0805 structures, and that has a confidence score of 0.24 [19]. This model predicted that the second sheet may have two additional strands, similar to the sheet composition of the bacterial enzyme, while the active site loop conformations were highly similar to the plant TRAcP rather than the bacterial metallohydrolase.

**Figure 5:**
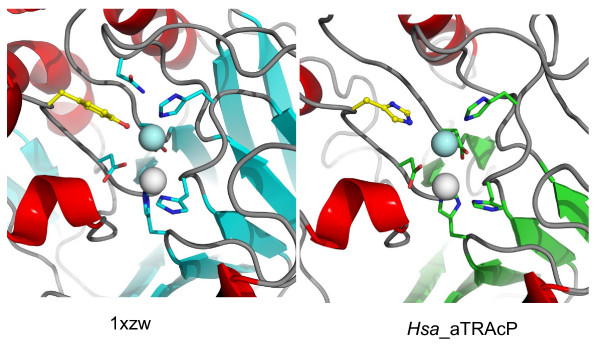
Structure of the modelled active site of *Hsa*_aTRAcP and that of 1XZW illustrating the difference in the Fe(III) coordination site. Substitution of the sweet potato TRAcP Tyr for His in *Hsa*_aTRACP (yellow, ball and stick representation) indicates that *Hsa*_aTRACP is not a member of the TRAcP family as the charge transfer transition required for the purple color is absent.

The model of *Hsa*_aTRACP reveals that the side chains of seven metal coordinating residues are likely to be spatially conserved in comparison to other binuclear metallohydrolases. The identity of six of the seven residues in *Hsa*_aTRACP are identical to that in TRAcPs with the exception that Tyr166 (sweet potato TRAcP numbering) is replaced by His93 in *Hsa*_aTRACP (Figure 5). Closer inspection of the model indicates that although Tyr219, 220 and 292 are located within a putative substrate binding site, they are not likely to form a charge transfer interaction with the metal ions in the active site. This places *Hsa_*aTRACP into a separate, nonpurple class of binuclear metallohydrolases with two soft metal binding sites that are likely favour the coordination of two divalent metal ions. A similar active site structure was reported for other members of the binuclear metallohydrolase family, notably Ser/Thr type protein phosphatases (PPs) such as the ones from bacteriophage λ (λPP) [20] and several mammalian organisms, i.e. one from rabbit (PP1) [21,22] and cow (PP2B) [23] and two from human (PP2B and PP5) [24], and more recently the Rv0805 cyclic nucleotide phosphodiesterase from *M. tuberculosis *[16]. PP2B is also known as calcineurin and plays a major role in the signal transduction cascade in T-cell activation [25]. The *in vivo *metal ion contents of PPs is not certain, but all are reported as M(II)-M(II) forms, where M = Fe, Zn or Mn [1].

## Conclusion

Previously we identified a high molecular weight human TRAcP [14]. Here we have extended this study through the characterisation of a second transcript, *Hsa*_aTRACP, that is a remote relative of the PAPs, sharing 18% sequence identity with the plant enzymes as the closest relatives with known structure. Analysis of the active site of *Hsa*_aTRACP indicates that it is not likely to be a purple protein due to the absence of an essential tyrosine ligand (Figure 5). In this respect, *Hsa*_aTRACP resembles some cyclic nucleotide phosphodiesterases and novel Ser/Thr PPs. This may therefore represent an event of divergent evolution in the binuclear metallohydrolase family. Based upon the pattern of expression and putative cytoplasmic location, we speculate that *Hsa*_aTRACP is another member of the cytoplasmic protein phosphatase family that is likely to have a role in the regulation of signalling.

## Abbreviations

PAP: purple acid phosphatase; PP: Ser/Thr protein phosphatase; TRAcP: tartrate-resistant acid phosphatase; rmsd: root mean square deviation.

## Competing interests

The authors declare that they have no competing interests.

## Authors' contributions

KSH generated homology model, performed database searches and contributed to manuscript writing. TH performed structure alignments. IC, LWG and DAH were interpreting data, designed experiments and provided critical feedback on the manuscript. JW, JR, AB and GK carried out cloning and expression experiments. GS and JUF were responsible for the conceptualization of experiments, analysis and interpretation of data, and drafting and critical review of manuscript.
